# Drinker Prototype Alteration and Cue Reminders as Strategies in a Tailored Web-Based Intervention Reducing Adults’ Alcohol Consumption: Randomized Controlled Trial

**DOI:** 10.2196/jmir.3551

**Published:** 2015-02-04

**Authors:** Britt van Lettow, Hein de Vries, Alex Burdorf, Brigitte Boon, Pepijn van Empelen

**Affiliations:** ^1^Erasmus MCDepartment of Public HealthRotterdamNetherlands; ^2^Maastricht UniversityDepartment of Health PromotionMaastrichtNetherlands; ^3^Trimbos InstituteDepartment of Public Mental HealthUtrechtNetherlands; ^4^TNOResearch Group Life StyleLeidenNetherlands

**Keywords:** Internet, intervention studies, prototypes, drinking, intention, willingness, adults, randomized controlled trial

## Abstract

**Background:**

Excessive alcohol use is a prevalent and worldwide problem. Excessive drinking causes a significant burden of disease and is associated with both morbidity and excess mortality. Prototype alteration and provision of a cue reminder could be useful strategies to enhance the effectiveness of online tailored interventions for excessive drinking.

**Objective:**

Through a Web-based randomized controlled trial, 2 strategies (ie, prototype alteration and cue reminders) within an existing online personalized feedback intervention (Drinktest) aimed to reduce adults’ excessive drinking. It was expected that both strategies would add to Drinktest and would result in reductions in alcohol consumption by intrinsic motivation and the seizure of opportunities to act.

**Methods:**

Participants were recruited online and through printed materials. Excessive drinking adults (N=2634) were randomly assigned to 4 conditions: original Drinktest, Drinktest plus prototype alteration, Drinktest plus cue reminder, and Drinktest plus prototype alteration and cue reminder. Evaluation took place at 1-month posttest and 6-month follow-up. Differences in drinking behavior, intentions, and behavioral willingness (ie, primary outcomes) were assessed by means of longitudinal multilevel analyses using a last observation carried forward method. Measures were based on self-reports.

**Results:**

All conditions showed reductions in drinking behavior and willingness to drink, and increased intentions to reduce drinking. Prototype alteration (B=–0.15, *P*<.05) and cue reminder usage (B=–0.15, *P*<.05) were both more effective in reducing alcohol consumption than when these strategies were not provided. Combining the strategies did not produce a synergistic effect. No differences across conditions were found regarding intentions or willingness.

**Conclusions:**

Although individuals’ awareness of their cue was reasonable, their reported alcohol consumption was nevertheless reduced. Individuals appeared to distance their self-image from heavier drinking prototypes. Thus, prototype alteration and cue reminder usage may be feasible and simple intervention strategies to promote reductions in alcohol consumption among adults, with an effect up to 6 months.

**Trial Registration:**

Nederlands Trial Register (NTR): 4169; http://www.trialregister.nl/trialreg/admin/rctview.asp?TC=4169 (Archived by WebCite at http://www.webcitation.org/6VD2jnxmB).

## Introduction

### Background

 Excessive alcohol use is a prevalent and worldwide problem [1]. In the Netherlands, 12.9% of the general population engages in weekly binge drinking, defined as ≥4 and ≥6 glasses of alcohol (10 gram each) per occasion for women and men, respectively. Also, 8.3% drink excessively, defined as drinking 14 or 21 glasses per week for women and men, respectively [2]. The percentage of drinkers and amount of alcohol consumed is generally higher among men than women [2]. Excessive drinking causes a significant burden of disease [3]. It is associated with both morbidity and excess mortality [4]. Also, it is an underlying cause, in part or entirely, of more than 30 health conditions and a contributing factor to many more problems, such as social harm, costs, etc [5].

It is important to further our understanding of how to reduce excessive drinking. A large number of interventions have targeted drinking behavior assuming that behavior is intentional. However, medium-to-large changes in intentions only lead to small-to-medium changes in behavior [6]. Effect sizes are found to vary for different behavior types and specific populations (eg, age-specific) with lower effect sizes for risk behavior than for health behavior [7]. A meta-analysis showed that, among the interventions that were based on Theory of Planned Behavior (TPB) components [8], only half were found to guide changes in intentions and two-thirds guided changes in behavior, and only small effect sizes were produced [9]. In addition, a meta-analysis based on 7 studies found a medium effect size (Hedges’ g=0.39) regarding the effectiveness of online self-help interventions in reducing adults’ drinking behavior in the general population, with an effect up to 6 or 9 months [10]. These types of interventions have several advantages, such as reach and cost-effectiveness (eg, [11-14]). However, single-session interventions, such as Drinktest.nl (described subsequently), have been found to produce small effect sizes only [10]. Drinktest has been shown to be more effective at reducing alcohol consumption among adult males in the experimental group than in the control group up to 1-month follow-up, but not up to 6-month follow-up [15]. In sum, the results of previous research and interventions often focused on explaining or changing intentional behavior; these suggest that a significant proportion of intentions and behavior remains unexplained and that the effectiveness of interventions can be improved.

Two main reasons may account for the small-to-medium (or lack of long-term) effects. First, individuals may not be fully aware of the opportunities of how to act on their intentions. For example, in the case of drinking behavior, the individual may intend to limit his alcohol consumption. The person needs to be aware of, for example, opportunities and resources to accomplish this limitation, such as responses to others to resist drinks when offered. As a result, many studies and interventions have focused on helping people act on their intentions (eg, [16]), acknowledging the well-known intention-behavior gap. However, the second reason is that behaviors may occur without intentions or even when having intentions not to do so [17,18]. Risk behaviors may also be guided by socially induced situations and factors, such as impulsivity, sensation seeking, and heat of the moment [19], following implicit and social reactive processes [20]. Importantly, people do not always comply with their intentions, and intentions are less likely to predict impulsive behaviors (eg, excessive drinking). Therefore, some researchers have suggested that targeting this social reactive process may be more fruitful than addressing the explicit goal-directed route to overcome these issues [21-23].

### Additional Strategies

This study addresses these issues by examining the effect of 2 intervention strategies that could potentially help enhance the effect of an existing online (ie, Web-based) tailored intervention, Drinktest.nl: prototype alteration and cue reminders. Drinktest is based on the TPB [8], I-Change [24], and Stages of Change Model [25], providing normative and personalized feedback regarding self-help guidelines to reduce alcohol consumption. As described previously [15]:

Drinktest was developed by the Netherlands Institute for Health Promotion and Disease Prevention (NIGZ). Drinktest offers brief personalized feedback regarding an individual’s personal alcohol consumption patterns. The intervention consists of various components: overview of mean weekly alcohol intake, associated health risks, self-help guidelines to reduce alcohol intake, and normative feedback to compare one’s own alcohol consumption to the level of one’s own cohort.

The first strategy that could potentially enhance the effect of Drinktest is prototype alteration. Prototypes refer to the mental image of a typical person engaging in a certain behavior [17,18], such as a typical drinker or smoker. Prototypes are described in the Prototype Willingness Model (PWM), a dual-process model [17,20,23] assuming that behavior is guided by (1) reasoned intentions and (2) unintentional implicit social reactions. These “routes” may coexist in guiding behavior. For unintentional implicit social reactions, behavior is the result of behavioral willingness (further referred to as willingness). Willingness is defined as an “openness” to risk situations [18,20], such as the willingness to drink more than was planned. Specifically, many risky behaviors are facilitated or prompted by external stimuli or (social) situations [18]. Thus, the PWM recognizes factors such as impulsivity.

Prototypes have been shown to explain behavior through their effect on willingness and intentions and have also been shown to directly explain drinking behavior [26-31]. The assumption is that the more similar to the self and the more favorably the prototype is perceived, the more the individual will be willing or intending to engage in certain behavior [17,20]. Prototypes can incorporate core values (ie, goal states) that individuals desire (or avoid) (eg, [18,32]). Altering the perception of prototypes can be used as a strategy to cultivate behavior change by, for instance, contemplation of or accentuating the negative or positive characteristics attributed to the prototypes [33,34] and by encouraging social comparison and distancing from health-risk prototypes [35,36]. Experiments and intervention studies revealed that prototype alteration was effective in (1) postponing the onset of drinking among children aged 10-12 years with an effect up to 2 years [37], (2) quitting success for an adult smoking cessation group [36,38], and (3) changing (health-risk and health-protective) behavior among adolescents and undergraduates [28,33,34,37,39]. Although prototype alteration has been applied to alcohol use, few interventions aimed at reducing excessive drinking by using dual-process models (PWM) have been applied to young adults (usually incorporating only university students) and results have been mixed [40-42]. To our knowledge, there are no such interventions for the general adult population.

A second strategy is the use of cue reminders. The limited number of studies focusing on cue reminders has shown that cue reminders can help in changing (and maintaining) behavior [43-45] because cue reminders can help people remember the content of interventions or their personal goals. Cue reminders can support enactment of intentions as they can unconsciously prompt self-enhancing or self-protecting opportunities. That is, experimental research suggests that cue reminders could function through their salience and through an inhibiting mechanism. This would result in the inhibition of other cues (ie, to engage in health-risk behavior) that are present in a situation and, as a result, impulsive behaviors can be hampered [43,44]. Cue reminders are found to be effective even when people lack the cognitive capacity to reason, such as when being under time pressure or when already having consumed alcohol. This suggests an effect through the implicit route [43,44]. Finally, a cue reminder strategy has the advantage that it can be a simple means, such as a bracelet (one’s own or provided), that can remind people of an intervention or of their intentions.

This study examined whether prototype alteration and provision of a cue reminder can be useful strategies to enhance the effectiveness of an existing online (ie, Web-based) tailored intervention, Drinktest. Drinking behavior, intentions to reduce drinking, and willingness to drink were targeted as primary outcomes. It was expected that (1) prototype alteration may intrinsically motivate people to drink less, (2) cue reminders may strengthen the salience of alcohol reduction goals, and (3) the combination of prototype alteration and a cue reminder may produce a synergistic effect and thus increase the salience and intrinsic motivation to drink less. As such, we tested whether the strategies of prototype alteration and a cue reminder in addition to the Drinktest intervention would be more effective in addressing excessive drinking behavior than the original Drinktest without those additional strategies. Other outcomes are also addressed, as will be described subsequently.

## Methods

### Design and Participants

A randomized controlled trial was conducted in the Netherlands in which participants were randomly assigned by computer to 1 of 4 conditions: (1) original Drinktest, (2) Drinktest plus prototype alteration, (3) Drinktest plus cue reminder, and (4) Drinktest plus prototype alteration and cue reminder (further referred to as the “combined condition”). The online tailored intervention consisted of baseline measurements and tailored feedback. Follow-up measurements were conducted at 1 and 6 months (postintervention: T2 and T3). Eligible participants were individuals aged 18 or older engaging in excessive drinking: exceeding ≥14 and ≥21 glasses of alcohol per week or drinking ≥4 and ≥6 glasses per occasion for women and men, respectively [46]. This norm was set by the original Drinktest and left unchanged.

### Recruitment and Procedure

Participants were recruited online and by printed materials (posters and newspaper advertisements) from September 2012 to June 2013. The website of Drinktest was also easily accessible by online search engines. Before entering the intervention (T1), participants read the study information and were told that the existing Drinktest website was being evaluated. It was explicitly stated that participants did not have to commit themselves to reducing their alcohol consumption. Participants were then asked to sign the online informed consent form. In case participants declined to participate, they could close the browser or receive the original Drinktest without taking part in the study. After the informed consent form had been signed, participants were randomized into the conditions. Nonexcessive drinkers (of which the status was known only after drinking behavior was measured) were excluded from the study sample and routed to the original Drinktest.

All questions were self-administered and data were collected online. Participants were invited by email to participate in the 2 follow-up measurements and received reminders if necessary (maximum of 3). Participants were invited for the 6-month follow-up irrespective of their participation in the 1-month follow-up. A total of 50 vouchers worth €50 were distributed (by means of a raffle) as incentive. Ethical approval was granted by the independent ethics committee of the Erasmus MC, Rotterdam, the Netherlands (ref no: MEC-2010-112).

Power analyses using G*Power3 (eg, [47]) were performed. We expected to need a total sample size of 368 (4 groups × 3 time repetitions ANOVA_rm (repeated measures); ES(f)=.10, alpha=.05, power=.80, nonsphericity correction=.50, rho=.5), excluding dropout. Compensating for dropout (25% was expected), approximately 480 (120 participants per subgroup) would be needed. Given the larger dropout than expected, a larger inclusion at baseline was needed and achieved. As a result, the minimal required criterion of 480 participants to be included in all 3 measurements was met.

### Intervention

#### Overview

**Figure 1:**
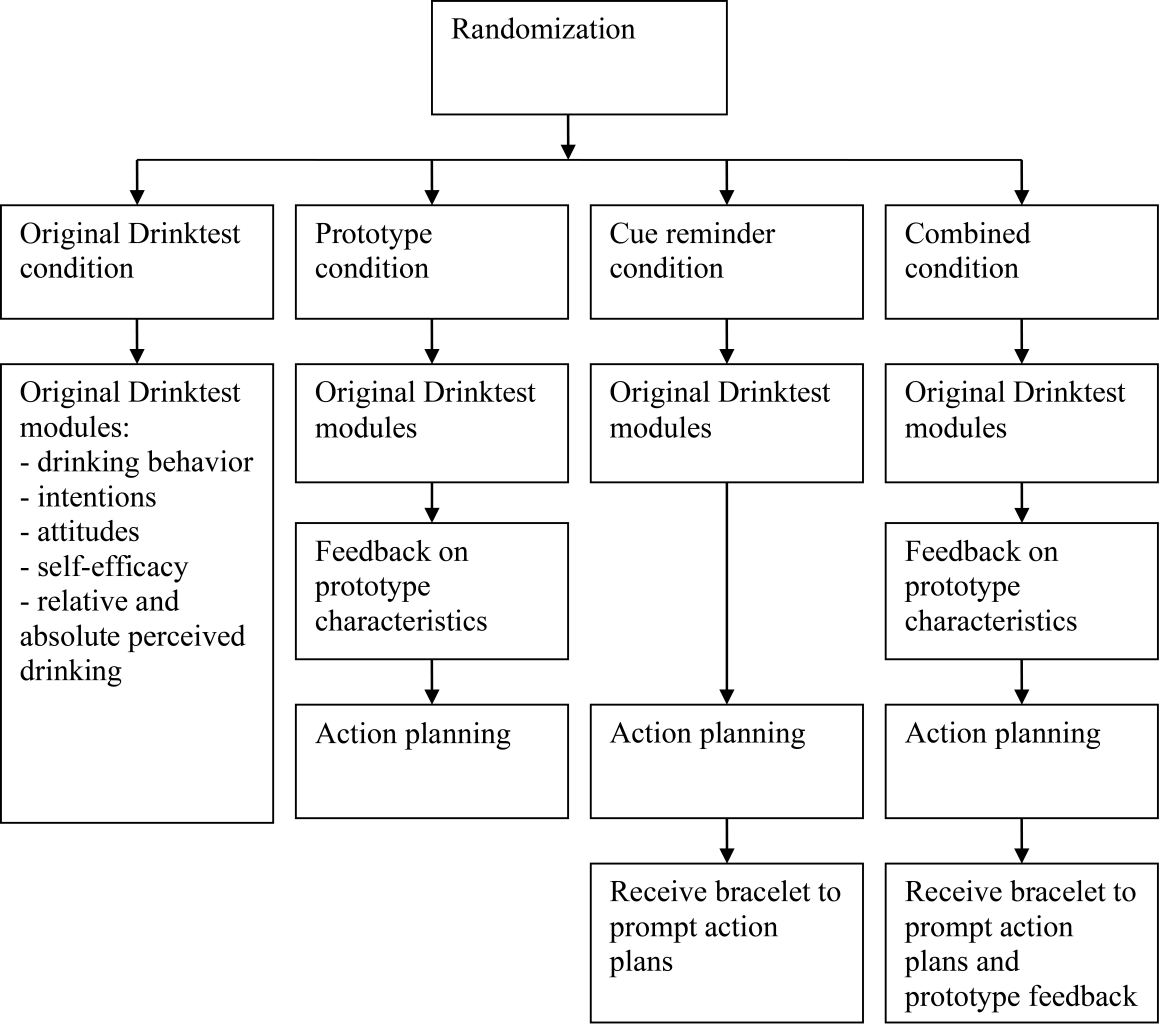
Flowchart illustrating the flow of the intervention per condition.

Figure 1 represents the flow of the intervention. All tailored feedback was based on participant’s responses and gender and was delivered online. All participants, irrespective of condition, received questions and feedback according to the original Drinktest. Feedback was derived from a computer program linking each possible combination of responses with an appropriate message. Feedback was not provided during the second and third measurement.

#### Original Drinktest Condition

Participants in the original Drinktest condition only received the standard version, in which they received feedback tailored to demographic background (gender), alcohol consumption, and intentions to reduce drinking. These messages reflected on personal drinking levels in comparison to the Dutch norm and peers’ drinking behavior, the correctness of their absolute and relative perceived drinking risks regarding health risks due to their alcohol consumption, intentions, temptations (eg, coping with fights), correctness of positive effects of alcohol (eg, whether alcohol helps to sleep better), and correctness of negative effects of alcohol (eg, consequences for the liver and heart). To improve self-efficacy participants were encouraged to make a plan (without guidance) or to balance the advantages and disadvantages of reducing alcohol consumption. This part took approximately 10 minutes [15]. Multimedia Appendix 1 provides examples.

#### Prototype Condition

After completing the original Drinktest, participants in the prototype condition received feedback regarding prototype alteration (see Measures and Figure 1, and see Multimedia Appendix 1 for examples) tailored to gender, drinking behavior (also including normative feedback), intentions, and prototypical self-characterization. This addition to the Drinktest took approximately 5 minutes. The prototype message reflected on characteristics that the participants evaluated as personally desirable or undesirable by evaluating oneself on 11 characteristics (see Measures). Negative characteristics were accentuated as being negatively valued by peers and were linked to excessive drinking (ie, implicitly referring to heavier drinking prototypes) and positive characteristics were linked to moderate drinking and being positive valued by peers (ie, moderate drinker prototype). Participants were encouraged to reduce their drinking to achieve their desired characteristics and, in turn, to be positively valued by peers. Thus, this feedback implicitly aimed to distance participants from the heavier drinking prototypes, such as the drunk and heavy drinker, and to encourage similarity to and favorability of the moderate drinker prototype (see [35,36,38]).

Then, participants were guided in their goal setting by selecting action plans (adapted from [48,49]) to achieve the desired characteristics. First, they selected how they felt about reducing their alcohol consumption after having received tailored feedback ranging from 1=“I do not wish to reduce my alcohol consumption” to 4=“I want to quit drinking.” If they were in doubt or were certain about reducing or quitting, participants were guided in their action plans by selecting a date to quit or start reducing. If they chose to reduce their consumption, they could set a limit of number of glasses per day and per week and the number of days on which the participant will not drink alcohol. Participants could also refuse to make plans (ie, “I do not wish to make a plan”) or could set their own goals. (Participants in the original Drinktest condition did not form action plans.)

Participants selected action plans rather than forming their own because (1) forming plans of good quality has proven to be difficult for participants [50] and (2) plans formed by individuals are subject to additional variables compared to plans provided by the researcher [51].

#### Cue Reminder Condition

After finishing the original Drinktest modules, cue condition participants followed the same procedure in forming action plans as in the prototype condition (adapted from [48,49]). Feedback was provided that reflected on their action plans explaining that a cue reminder may help remember their plans (if made) and they were offered a free silicone bracelet (see [43]) by mail. If participants did not want to receive the bracelet, they were encouraged to select a piece of their own jewelry or another object of frequent use. After the cue selection, participants were instructed to think of their plans when they were aware of their cue so that the cue was linked to the action plans. If no plans were formed, participants were requested to use a cue for the duration of 1 month for the sake of the study and they were told to think of the content of Drinktest when they were aware of the cue. All participants were asked to wear their cue at least 1 month (ie, until T2). See Multimedia Appendix 1 for examples. This addition to the Drinktest took less than 5 minutes.

#### Combined Condition

Participants in the combined condition completed the original Drinktest modules, the prototype alteration module, and the cue module (see Figure 1). These participants were offered a cue reminder and were instructed to remember their plans (if made) and the desired characteristics they could achieve by reducing their alcohol consumption when they were aware of the cue reminder. See Multimedia Appendix 1 for examples.

### Measures

#### Overview

All measurements included the same questions and followed the same guidelines for drinking norms unless otherwise specified. Measures from the original Drinktest were left unchanged and items regarding demography, willingness, prototypes, cue reminder, and process evaluation were added.

#### Process Evaluation (Measured at T2)

Participants reported on their appreciation of the intervention at the 1-month posttest by answering the statement: “The information and advice of Drinktest.nl were...” Answers ranged from 1=I disagree to 7=I agree regarding reliability, novelty, being informative, ease of understanding, personal relevance, persuasiveness, enjoyability, and usefulness (α=.86).

At 1-month posttest, all participants were asked, regarding the past 4 weeks (1) how aware they had been of their alcohol use, (2) how often they had contemplated on the intervention’s feedback, and (3) their perception of having tried to reduce their alcohol consumption. Finally, we checked whether participants had correctly remembered their choice of cue, how aware they were of their cue, and how often they had worn or used the cue reminder. Answers to the Likert scales ranged from 1=not at all to 7=a lot.

#### Self-Characterization (Measured at T1, T2, and T3)

These items were assessed only at baseline among the prototype and combined conditions because it was part of their manipulation and feedback. Participants were asked to characterize themselves by prototypical characteristics. That is, prototypes are usually assessed by a list of characteristics describing them (eg, [18,52]). In this case, participants were instructed to rate themselves (ie, self-image) on 11 semantic pairs of prototype adjectives to reflect which adjectives they generally desired to be described with (7-point scale). The adjectives (ie, characteristics) were derived from a previous study on drinker prototypes [53]: unsociable-sociable, insecure-self-confident, loud-quiet, volatile-nonvolatile, reserved-spontaneous, annoying-funny, boring-amiable, sad-cheery, uncontrolled-controlled, irresponsible-responsible, and unordered-determined. A higher mean indicated a more positive desired self-image (T1-T3: α=.79-.86).

#### Primary Outcome Measures (Measured at T1, T2, and T3)

##### Drinking Behavior

Drinking behavior was assessed by the Dutch version of the Quantity-Frequency-Variability (QFV) index of alcohol intake [54], which asked participants to report the number of glasses they had consumed for each day of the past week. The mean number of drinks per day was calculated and used for analyses. A standard unit of alcohol contains 10 gram of ethanol, generally irrespective of the type of drink.

##### Intentions

To assess intentions, the item was framed by Drinktest in behavioral stages in which participants chose from the following options: (1) I do not plan to reduce my alcohol consumption, (2) I plan to reduce my alcohol consumption within half a year, (3) I plan to reduce my alcohol consumption within a month, (4) I already started reducing my alcohol consumption, and (5) I have reduced my alcohol consumption more than half a year ago. This single item was treated as a continuous variable.

##### Behavioral Willingness

Willingness was assessed by describing a scenario with 2 possible actions (adapted from [22,42]): “Imagine that it is Saturday night. You’re going out with friends and you already had several alcoholic drinks. You feel you’ve had enough. One of your friends offers you a drink.” This scenario was followed by the question “How willing would you be to...” with the statements “I take it and drink it” and “I refuse” rated from 1=certainly not to 7=very certain (T1-T3: *r*=.76-.85). Answers to the second statement were reversed.

#### Secondary Outcome Measures (Measured at T1, T2, and T3)

##### Absolute and Relative Perceived Drinking

Absolute perceived drinking risks was assessed with the item “With regard to my health, I consume too much alcohol” rated from 1=I disagree to 3=I agree. Relative perceived drinking was assessed with the item “Compared to [women/men] of my age, I drink...” rated from 1=a little to 3=a lot.

##### Attitude

Attitude was examined by the original Drinktest using 12 items measuring advantages and disadvantages of drinking alcohol regarding health, sociability, and coping. For instance, “My alcohol use is healthy for my heart and veins” rated from 1=yes, healthy to 3=no, unhealthy and “My alcohol use is a bad example to others” and “My alcohol use is bad for my liver” both rated from 1=yes, bad to 3=no, good. If needed, items were reversed so that a higher score represented a more positive attitude toward drinking. Because reliability over the 12 items was low, principle component analysis was performed revealing 2 factors. Only the first factor (5 items regarding relaxation, sleep, group conformation, sociability, and coping) was used in analyses (T1-T3: α=.73-.78) because the second factor still had low reliability (T1-T3: α=.35-.43).

##### Self-Efficacy

A single item assessed self-efficacy: “I find reducing my alcohol use” rated from 1=very hard to 5=very easy.

##### Temptations

Twelve items examined temptations, which regarded emotions, coping, habit, and social situations, such as “How tempting do you find it to drink alcohol when you are at a party or in a restaurant?” with answers ranging from 1=not tempting at all to 5=very tempting (T1-T3: α=.86-.87).

### Statistical Analyses

All analyses were performed in SPSS 21.0 (IBM Corp, Armonk, NY, USA). First, we determined whether dropout between baseline and follow-up measurements was different for condition, gender, age, ethnicity, level of education, intentions, willingness, and drinking behavior. Second, potential differences between conditions at baseline were assessed regarding these measures. Third, the process evaluations were assessed. Fourth, longitudinal multilevel analyses (mixed models) were performed using the last observation carried forward (LOCF) method (1) to account for dropout and (2) because of the nested design (measurements such as time were nested in individuals). Using the LOFC method implies that if data for a follow-up measurement were missing, then data from the previous known data were used for analyses. For example, if data were available for the first and third measurements and the second was missing, then the data from the first measurement were also used as the second measurement instead of treating this data as missing. It should be noted that reported descriptives are based on LOCF.

Following previous research [55], a multilevel regression model for longitudinal data was used including a random slope and intercept to analyze differences between conditions in the changes in the dependent variables from baseline to both follow-up measurements. The most important reason for using this method is that it provides a solution to the problem of missing data and thereby increases the power of the study [56].

The following independent variables were included in the multilevel longitudinal analyses: having received prototype alteration feedback (yes/no), having received a cue reminder (yes/no), and the interaction of prototype alteration (yes/no) and cue reminder (yes/no) to assess the added value of their combination (following previous research [57]; between-group variable), and including time (measurements, coded as 0, 1, 2 following Blom et al [55]; within-participant variable). For instance, the analysis group that received the prototype alteration strategy (prototype=yes) was compared to the group that did not receive a prototype alteration strategy in addition to the Drinktest (prototype=no). Analyses were corrected for potential significant differences between conditions at baseline. The means are given per analysis group instead of per condition for clarity and continuity with the effects presented in the tables and figures. For sensitivity purposes, the analyses were repeated for complete cases only. We used the median absolute deviation (MAD) to detect outliers for the behavioral measures (at all time measurements). MAD was applied because it is more robust to outliers than standard deviation [58]. After applying MAD, the variables were normally distributed.

## Results

### Participants’ Characteristics

**Figure 2:**
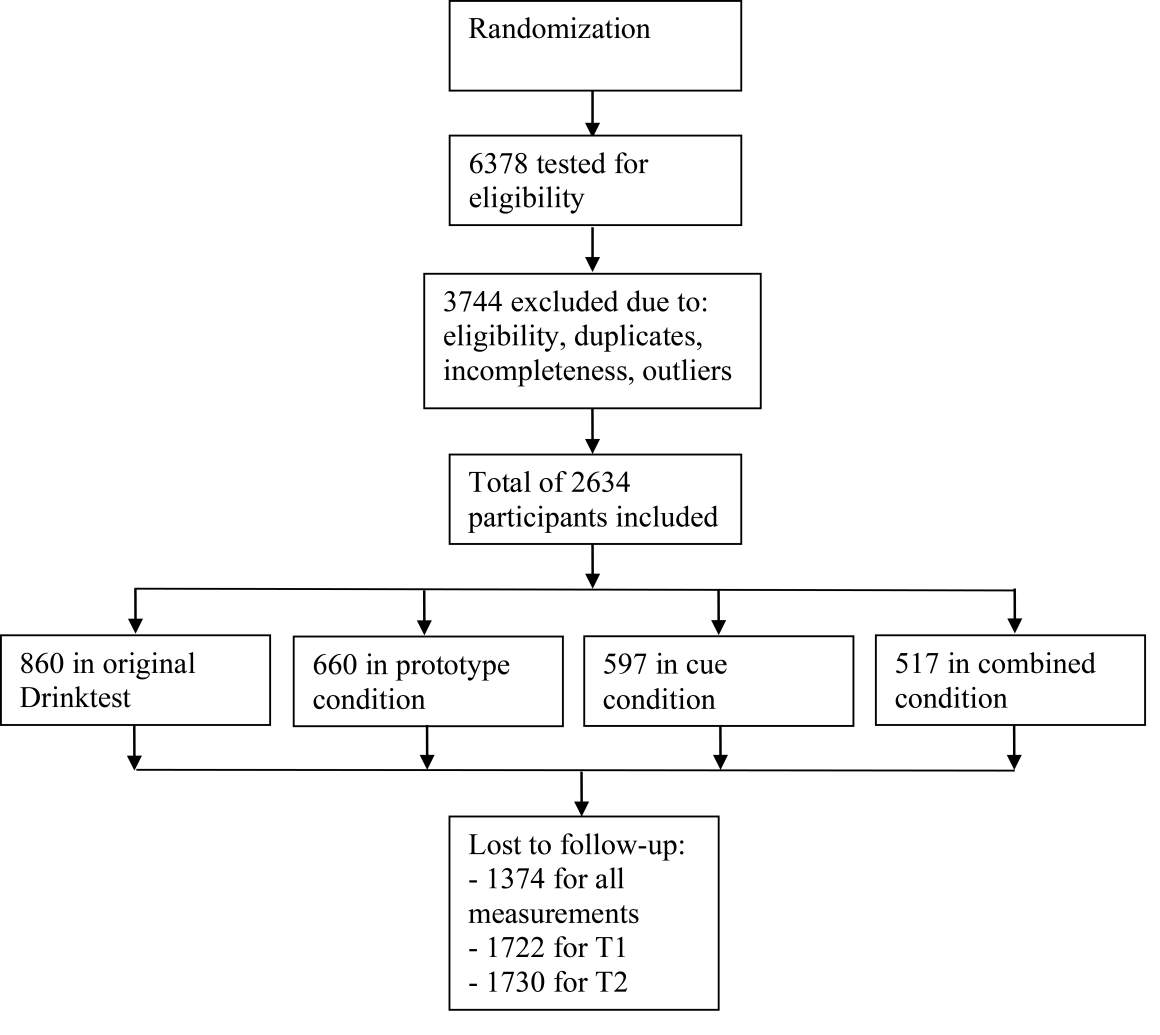
Flowchart illustrating the flow of participants through the study.

Figure 2 presents the flowchart of participants showing that a total of 6378 persons started the program. After data collection was completed, 9 same email addresses were used by different persons and were removed (n=19). Then, duplicates (n=99), nonexcessive drinkers (n=2506), incomplete (n=892), and outliers based on MAD (n=228) at baseline were removed. The resulting final sample consisted of 2634 eligible participants (male: 1351/2634, 51.29%; age: mean 37.03, SD 15.19). LOCF was applied. Most (94.46%, 2488/2634) of the sample was of Western origin, as defined by Statistics Netherlands [59], most originating from the Netherlands, followed by Belgium and Germany. *Western origin* includes all countries in Europe (except for Turkey), North America, Oceania, Japan, and Indonesia (including former Netherlands East Indies). *Non-Western* includes Turkey and all countries of Africa, Latin America, and Asia, except Japan and Indonesia [59]. Also, most were either pursuing or had completed a middle or higher educational level (64.58%, 1701/2634).

Intervention analyses were corrected for age and educational level because these were significantly different between conditions at baseline. Table 1 presents the baseline characteristics of participants overall and per condition.

**Table 1:** Participant characteristics and primary outcome measures at baseline (T1) presenting differences between study conditions.

Variables		Condition				Overall (N=2634)	*F*_3,2633_	χ^2^_3_	*P*
		Original Drinktest (n=860)	Prototype (n=660)	Cue reminder (n=597)	Combined (n=517)				
Age (years), mean (SD)		35.24 (15.30)	37.43 (15.03)	37.43 (15.03)	39.03 (15.18)	37.03 (15.19)	7.33		<.001
**Gender, n (%)**								4.6	.20
	Male	467 (54.3)	330 (50.0)	297 (49.7)	257 (49.7)	1351 (51.29)			
	Female	393 (45.7)	330 (50.0)	300 (50.3)	260 (50.3)	1283 (48.71)			
**Educational level**								15.4	.002
	Low	346 (40.4)	224 (34.0)	197 (33.1)	161 (31.1)	928 (35.23)			
	High	511 (59.6)	435 (66.0)	399 (66.9)	356 (68.9)	1701 (64.58)			
**Origin, n (%)**								0.4	.94
	Non-Western	49 (5.7)	33 (5.0)	31 (5.2)	28 (5.4)	141 (5.35)			
	Western	809 (94.3)	626 (95.0)	564 (94.8)	489 (94.6)	2488 (94.46)			
Drinking behavior, mean (SD)		3.51 (1.82)	3.65 (1.79)	3.64 (1.83)	3.64 (1.83)	3.60 (1.82)	1.08		
Intentions, mean (SD)		2.58 (1.40)	2.71 (1.34)	2.69 (1.36)	2.71 (1.33)	2.66 (1.36)	1.58		
Willingness, mean (SD)		4.60 (1.98)	4.65 (1.95)	4.57 (2.04)	4.41 (2.08)	4.57 (2.01)	1.57		

### Dropout

A total of 1260 participants completed 1 or both of the follow-up measurements (attrition 47.84%, 1260/2634). A total of 599 participants participated in all 3 measurements (attrition 77.26%, 599/2634). Dropout analyses were performed for those who did not participate in either of the 2 follow-up measurements. Dropout was highest among the original Drinktest condition (57.4%, 494/860) and was significantly higher than the prototype condition (OR 1.48, 95% CI 1.20-1.81, *P*<.001), cue condition (OR 1.26, 95% CI 1.02-1.55, *P*=.03), and combined condition (OR 1.38, 95% CI 1.10-1.71, *P*=.004); the 3 added conditions did not differ from one another. Dropout was also higher among men (OR 1.34, 95% CI 1.15-1.57, *P*<.001), lower educated participants (OR 2.21, 95% CI 1.87-2.60, *P*<.001), and non-Western participants (OR 1.46, 95% CI 1.03-2.07, *P*=.03). Additionally, those who dropped out were also slightly younger (*F*_1,2633_=48.83, *P*<.001) and reported a slightly higher alcohol consumption (*F*_1,2633_=17.66, *P*<.001). We used LOCF in the longitudinal multilevel analyses to account for dropout and corrected the analyses for age and education.

### Process Evaluation

Second, the appreciation of the intervention was assessed. The original (mean 4.85, SD 0.96) and extended Drinktest (combining the 3 added conditions; mean 4.88, SD 1.12) did not differ in their intervention evaluations (*F*_1,802_=0.06, *P*=.81). Both Drinktest versions were rated as equally interesting, new, informative, understandable, personally relevant, persuasive, enjoyable, and useful. The results were similar across all 4 conditions.

Furthermore, among the participants in the cue and combination conditions, 34.2% (193/564) received a bracelet and 43.1% (243/564) chose to use their own cue, whereas only 22.7% (128/564) did not wish to be reminded. At follow-up, the vast majority were found to remember their chosen cue reminder correctly (94.1%, 365/388) and reported using or wearing their cue reminder frequently (61.4%, 127/207). The awareness of the cue was reasonable (mean 3.27, SD 2.11).

Based on the means, participants that received a prototype alteration and/or cue reminder strategy generally had higher awareness of their alcohol consumption, contemplation of the intervention, and perception of having reduced alcohol consumption than those who only received the original Drinktest (Table 2). For those in the combination condition, a significant higher contemplation of the intervention was found compared to those in the original Drinktest condition. Also, participants in either the prototype or combination condition reported higher perceptions of having tried to reduce their drinking than participants in the Drinktest condition. Furthermore, an increase of self-characterization was found for those participants that received the prototype alteration strategy.

**Table 2:** Means and standard deviation of process evaluation for 1-month posttest (T2) and 6-month (T3) follow-up measurements overall and per condition.

Variable		Condition, mean (SD)^a^				Overall, mean (SD) (N=2634)
		Original Drinktest (n=860)	Prototype (n=660)	Cue reminder (n=597)	Combined (n=517)	
Process evaluation						
**Awareness of drinking**						
	T2 posttest	5.33 (1.56)	5.64 (1.44)	5.51 (1.51)	5.65 (1.43)	5.53 (1.49)
**Contemplation of intervention**						
	T2 posttest^b^	2.83 (1.91)	3.23 (2.05)	3.28 (1.96)	3.70 (2.18)	3.23 (2.04)
**Tried to reduce drinking**						
	T2 posttest^c^	4.49 (2.09)	5.00 (2.09)	4.95 (2.13)	5.22 (2.11)	4.89 (2.12)
**Self-characterization**						
	T1 baseline		5.42 (0.92)		5.46 (0.87)	
	T2 posttest		5.57 (0.86)		5.66 (0.83)	
	T3 follow-up		5.63 (0.84)		5.73 (0.82)	

^a^ There were only means for self-characterization at baseline for the prototype and combination condition. Differences for contemplation and trying to reduce drinking are significant at *P*<.05. Analyses were corrected for age and level of education.

^b^ Original Drinktest and Combined differ.

^c^ Original Drinktest and Prototype differ, and Original Drinktest and Combined differ.

### Primary Outcomes

**Figure 3:**
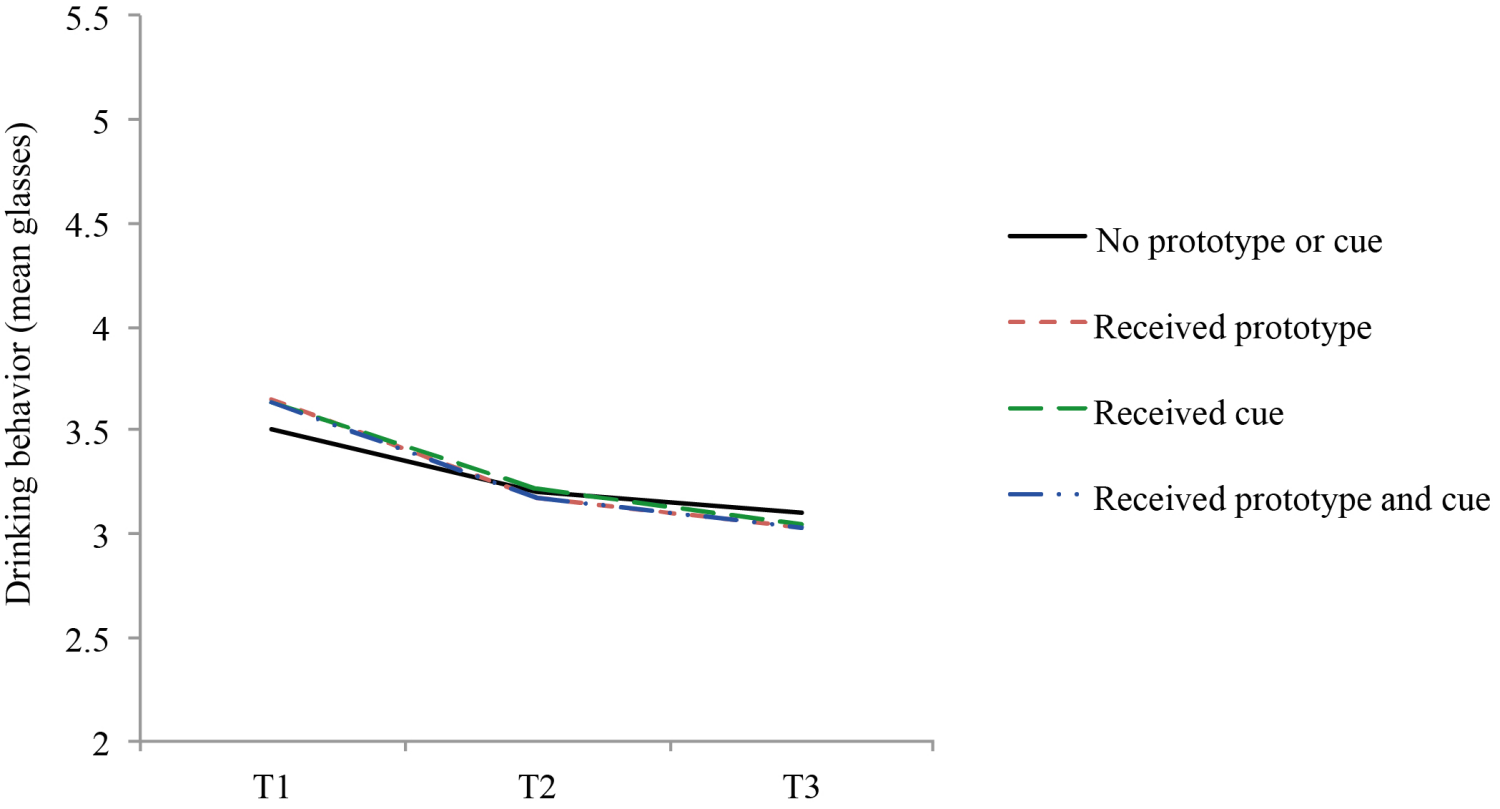
Effects on drinking behavior (mean glasses per day) per analysis group at baseline (T1), 1-month posttest (T2), and 6-month follow-up (T3).

Table 3 shows that the reported mean number of drinks per day was 3.60 glasses at baseline (SD 1.82), 3.19 glasses at 1-month posttest (SD 1.82), and 3.06 at 6-month follow-up (SD 1.81). Table 4 presents effects over time and for short-term (baseline and 1-month posttest) and long-term effects (baseline and 6-month follow-up). Alcohol consumption was reduced overall and participants who received the separate strategies of prototypes alteration (B=–0.15, *P*=.03) and a cue reminder (B=–0.15, *P*=.03) had larger reductions than those who did not receive these strategies in addition to the original Drinktest (Figure 3). The short-term effect was strongest and the long-term effect was only significant for the overall analysis (Table 4). Small effect sizes were found (Table 3).

**Table 3:** Means and standard deviations (based on last observation carried forward) and effect size (Cohen’s *d*)^a^ for primary and secondary outcomes for baseline (T1), 1-month posttest (T2), and 6-month follow-up (T3) measurements, overall and per analysis group.

Variable			No prototype or cue, mean (SD) (n=857)	Received prototype, mean (SD) (n=1176)	*d*	Received cue, mean (SD) (n=1113)	*d*	Received cue and prototype, mean (SD) (n=517)	*d*	Overall, mean (SD) (N=2634)
**Primary outcomes**										
	**Drinking behavior**									
		T1 baseline	3.51 (1.82)	3.65 (1.81)		3.64 (1.83)		3.64 (1.83)		3.60 (1.82)
		T2 posttest	3.20 (1.79)	3.17 (1.85)	0.09	3.21 (1.85)	0.07	3.18 (1.88)	0.08	3.19 (1.82)
		T3 follow-up	3.10 (1.81)	3.03 (1.82)	0.12	3.04 (1.84)	0.10	3.03 (1.88)	0.11	3.06 (1.81)
	**Intentions**									
		T1 baseline	2.58 (1.40)	2.71 (1.34)		2.70 (1.35)		2.71 (1.33)		2.66 (1.36)
		T2 posttest	2.67 (1.42)	2.86 (1.35)	0.04	2.83 (1.35)	0.03	2.85 (1.35)	0.04	2.79 (1.38)
		T3 follow-up	2.74 (1.45)	2.88 (1.41)	0.01	2.88 (1.43)	0.01	2.88 (1.43)	0.01	2.84 (1.43)
	**Behavioral willingness**									
		T1 baseline	4.60 (1.98)	4.55 (2.01)		4.50 (2.06)		4.41 (2.08)		4.57 (2.01)
		T2 posttest	4.45 (2.02)	4.25 (2.08)	0.08	4.24 (2.11)	0.06	4.11 (2.15)	0.08	4.34 (2.06)
		T3 follow-up	4.39 (2.04)	4.16 (2.06)	0.09	4.11 (2.11)	0.09	4.02 (2.10)	0.09	4.24 (2.07)
**Secondary outcomes**										
	**Attitude**									
		T1 baseline	1.48 (0.35)	1.47 (0.32)		1.46 (0.32)		1.46 (0.31)		1.47 (0.33)
		T2 posttest	1.47 (0.34)	1.45 (0.32)	0.03	1.46 (0.33)	–0.03	1.45 (0.31)	0.00	1.46 (0.33)
		T3 follow-up	1.46 (0.34)	1.46 (0.33)	–0.03	1.47 (0.34)	–0.09	1.47 (0.32)	–0.09	1.47 (0.34)
	**Self-efficacy**									
		T1 baseline	2.38 (0.96)	2.31 (0.93)		2.29 (0.92)		2.35 (0.94)		2.31 (0.93)
		T2 posttest	2.48 (0.94)	2.47 (0.98)	0.06	2.44 (0.95)	0.05	2.50 (0.96)	0.05	2.45 (0.96)
		T3 follow-up	2.58 (1.00)	2.54 (1.00)	0.03	2.48 (0.98)	–0.01	2.53 (0.97)	–0.02	2.53 (1.00)
	**Temptations**									
		T1 baseline	2.27 (0.43)	2.28 (0.42)		2.29 (0.42)		2.25 (0.42)		2.29 (0.42)
		T2 posttest	2.22 (0.43)	2.22 (0.42)	0.02	2.24 (0.42)	0.00	2.20 (0.43)	0.00	2.23 (0.42)
		T3 follow-up	2.48 (0.66)	2.44 (0.66)	–0.12	2.43 (0.66)	–0.16	2.42 (0.65)	–0.09	2.45 (0.66)
	**Absolute perceived drinking risks**									
		T1 baseline	2.38 (0.75)	2.47 (0.73)		2.49 (0.71)		2.48 (0.72)		2.45 (0.73)
		T2 posttest	2.37 (0.76)	2.81 (1.26)	0.47	3.27 (1.54)	1.05	3.27 (1.57)	10.07	2.77 (1.23)
		T3 follow-up	2.34 (0.77)	2.54 (1.06)	0.15	2.74 (1.28)	0.39	2.71 (1.30)	0.36	2.53 (1.03)
	**Relative perceived drinking**									
		T1 baseline	4.80 (1.25)	5.03 (1.15)		5.03 (1.18)		5.00 (1.16)		4.96 (1.20)
		T2 posttest	3.55 (1.53)	3.52 (1.61)	0.21	3.66 (1.66)	0.10	3.54 (1.62)	0.17	3.59 (1.61)
		T3 follow-up	3.21 (1.49)	3.13 (1.55)	0.25	3.23 (1.61)	0.17	3.16 (1.57)	0.20	3.19 (1.55)

^a^ Effect sizes (Cohen’s *d*) are based on changes between T1 and T2, and T1 and T3 for the analysis groups that received an additional strategy compared to the analysis group that did not receive an additional strategy added to Drinktest.

**Table 4:** Longitudinal multilevel analyses (mixed models) including analyses over all measurements (baseline, T1; 1-month posttest, T2; and 6-month follow-up, T3) and separately for short term (T1 to T2) and long-term measurements (T1 to T3), corrected for education and age. Regression coefficient (B) and 95% confidence intervals are presented regarding change over time for the strategy-added group versus no strategy added to the original Drinktest analyses group.

Variables and time^a^			Prototype received vs no prototype received		Cue reminder received vs no cue received		Interaction cue and prototype vs no additional strategy received		Effect over 4 conditions	
			B	95% CI	B	95% CI	B	95% CI	B	95% CI
**Primary outcomes**										
	**Drinking behavior**									
		T1,T2,T3	–0.15*	–0.28,–0.01	–0.15*	–0.29,–0.01	0.08	–0.11, 0.27	–0.07**	–0.11,–0.02
		T1,T2	–0.32*	–0.59,–0.05	–0.30*	–0.58,–0.02	0.27	–0.13, 0.66	–0.11*	–0.20,–0.02
		T1,T3	–0.22	–0.48, 0.05	–0.23	–0.50, 05	0.08	–0.31, 0.47	–0.11*	–0.20,–0.02
	**Intentions**									
		T1,T2,T3	–0.01	–0.10, 0.09	0.09	–0.01, 0.20	–0.08	–0.22, 0.06	0.01	–0.02, 0.05
		T1,T2	0.15	–0.05, 0.36	0.20	–0.01, 0.41	–0.18	–0.47, 0.12	0.06	–0.01, 0.13
		T1,T3	–0.06	–0.26, 0.14	0.15	–0.06, 0.36	–0.12	–0.41, 0.18	0.01	–0.06, 0.08
	**Willingness**									
		T1,T2,T3	–0.05	–0.18, 0.08	–0.10	–0.24, 0.05	0.01	–0.19, 0.21	–0.05	–0.09, 0.00
		T1,T2	–0.23	–0.51, 0.05	–0.12	–0.42, 0.17	–0.01	–0.42, 0.40	–0.10*	–0.19,–0.00
		T1,T3	–0.05	–0.32, 0.23	–0.17	–0.46, 0.12	0.02	–0.39, 0.43	–0.07	–0.16, 0.02
**Secondary outcomes**										
	**Attitude**									
		T1,T2,T3	0.00	–0.03, 0.03	0.01	–0.02, 0.04	0.00	–0.03, 0.04	0.00	–0.00, 0.01
		T1,T2	–0.03	–0.08, 0.03	0.01	–0.05, 0.07	0.01	–0.06, 0.08	0.00	–0.02, 0.02
		T1,T3	0.00	–0.05, 0.05	0.02	–0.04, 0.07	0.01	–0.07, 0.08	0.01	–0.01, 0.02
	**Self-efficacy**									
		T1,T2,T3	0.01	–0.06, 0.09	–0.02	–0.10, 0.06	0.01	–0.10, 0.11	–0.00	–0.03, 0.03
		T1,T2	0.00	–0.15, 0.16	0.04	–0.13, 0.20	0.03	–0.19, 0.24	0.02	–0.03, 0.08
		T1,T3	0.02	–0.13, 0.17	–0.05	–0.21, 0.11	0.02	–0.20, 0.23	–0.01	–0.06, 0.05
	**Temptations**									
		T1,T2,T3	–0.00	–0.06, 05	–0.01	–0.07, 0.04	–0.00	–0.07, 0.07	–0.01	–0.03, 0.01
		T1,T2	0.01	–0.10, 0.13	0.05	–0.06, 0.17	–0.08	–0.23, 0.06	0.00	–0.04, 0.04
		T1,T3	–0.00	–0.11, 0.11	–0.03	–0.15, 0.08	0.03	–0.11, 0.18	–0.01	–0.05, 0.04
	**Absolute perceived drinking risks**									
		T1,T2,T3	–0.01	–0.10, 0.09	0.27***	0.17, 0.37	–0.05	–0.17, 0.08	0.09***	0.06, 0.13
		T1,T2	0.00	–0.14, 0.15	2.46***	2.31, 2.61	–0.27***	0.48,–0.07	0.90***	0.85, 0.95
		T1,T3	–0.01	–0.15, 0.13	–0.10	–0.24, 0.05	–0.04	–0.24, 0.17	–0.05	–0.10, 0.00
	**Relative perceived drinking**									
		T1,T2,T3	–0.11*	–0.22,–0.01	–0.13*	–0.24,–0.02	0.04	–0.09, 0.18	–0.07**	–0.10,–0.02
		T1,T2	–0.17*	–0.33,–0.01	–0.12	–0.29, 0.04	0.04	–0.18, 0.26	–0.07*	–0.13,–0.01
		T1,T3	–0.23**	–0.39,–0.07	–0.21*	–0.38,–0.05	0.16	–0.07, 0.38	–0.09**	–0.15,–0.03

^a^ T1,T2,T3 refers to analyses showing whether there is an effect over time during all 3 measurements for the added strategy vs no added strategy; T1,T2 represents short-term effects from T1 baseline to T2 posttest; T1,T3 represents long-term effects from baseline T1 to T3 follow-up.

*P<.05, **P<.01, ***P<.001.

On average, intentions to reduce alcohol consumption increased and behavioral willingness to drink more decreased over time (Table 3), but no differences were found across groups (Table 4). An exception was a significant short-term overall effect on willingness. The interaction of prototype alteration × cue reminder was not significant; thus, it did not produce an extra effect beyond the effect of the separate strategies.

### Secondary Outcomes

Although a change in attitude, temptation, and self-efficacy was found (see means in Table 3 and effects in Table 4), prototype alteration or a cue reminder did not produce a larger change than when those strategies were not received.

**Figure 4:**
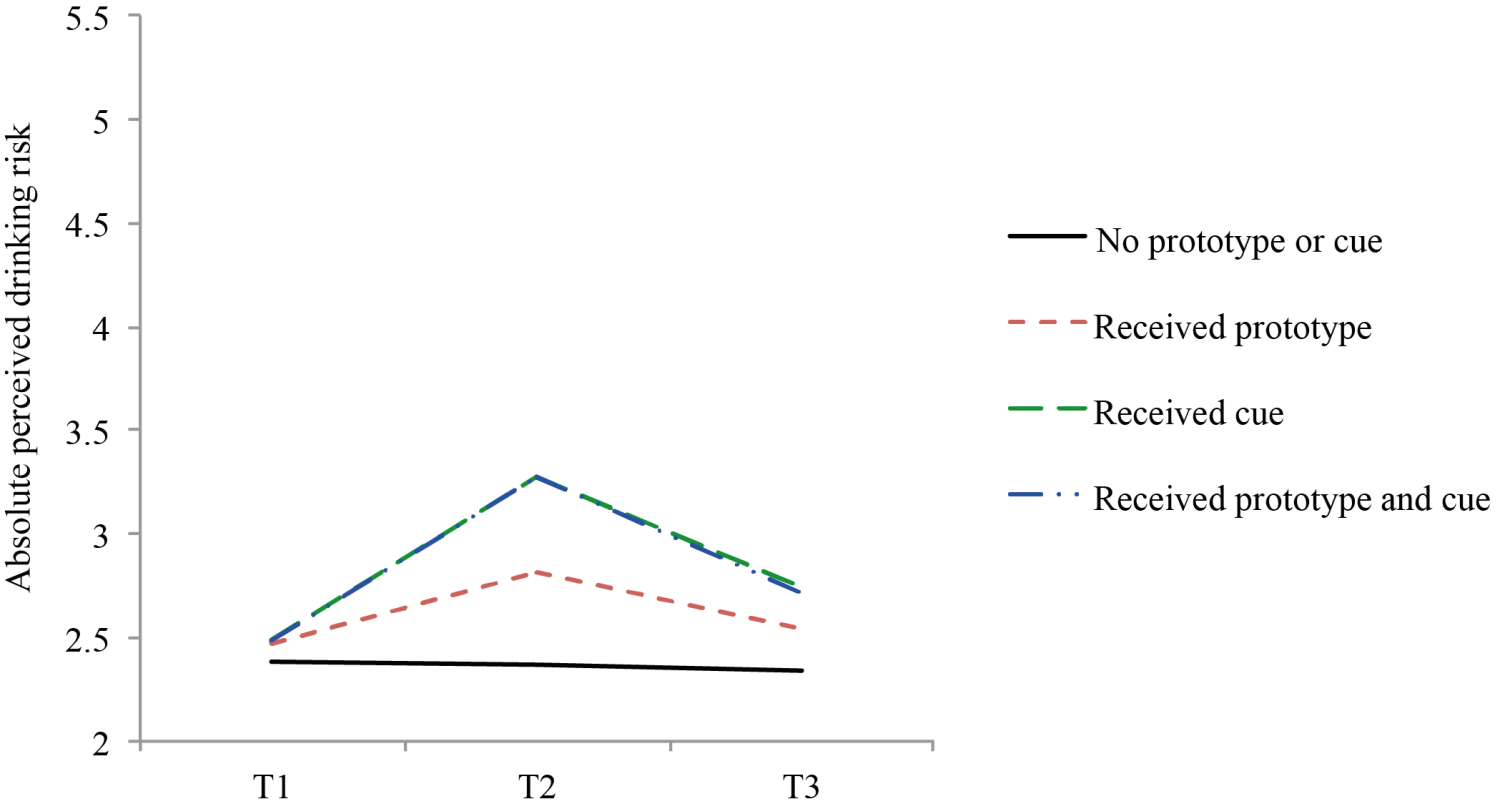
Effects on absolute perceived drinking risks per analysis group (means) at baseline (T1), 1-month posttest (T2), and 6-month follow-up (T3).

**Figure 5:**
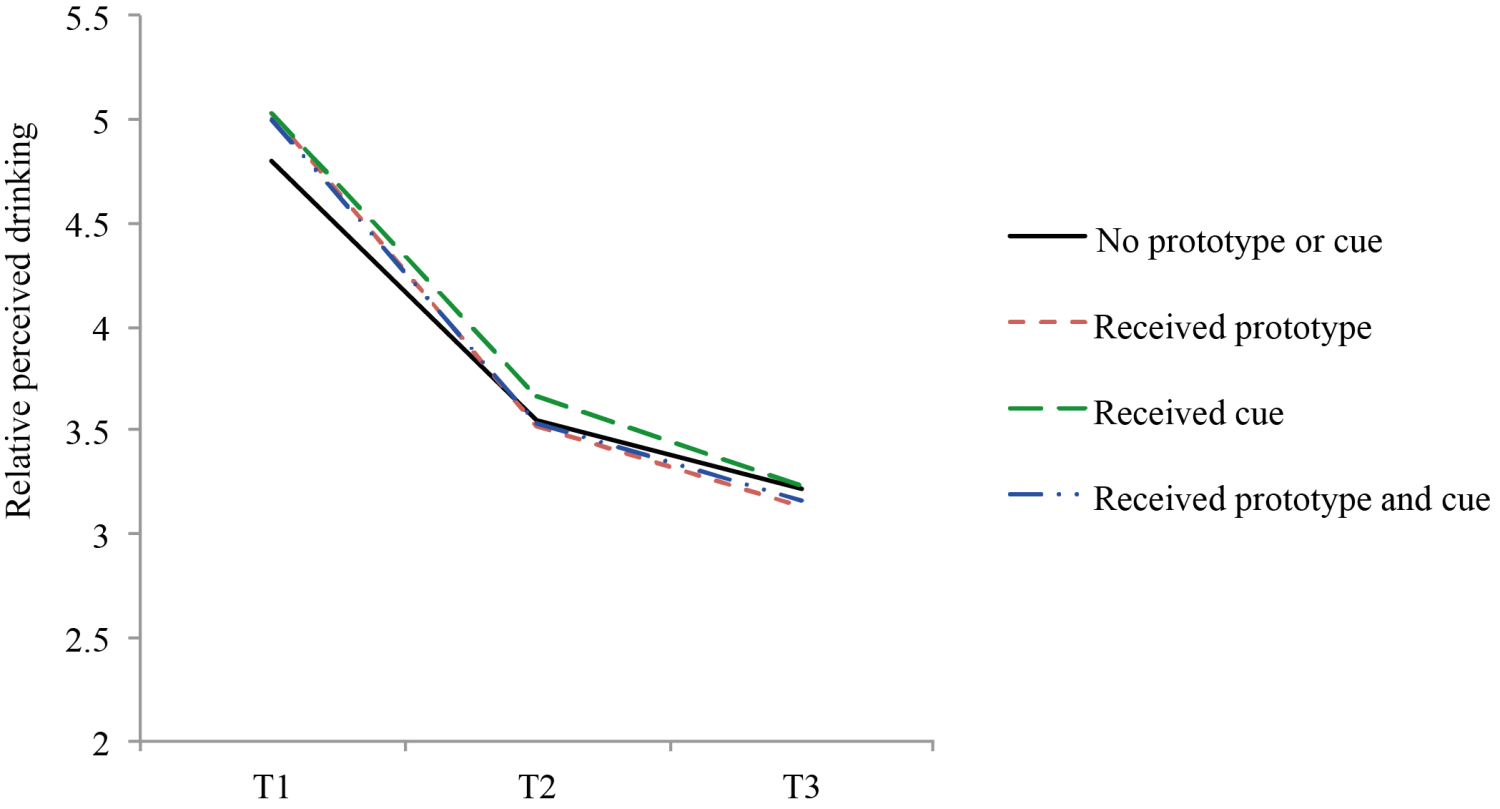
Effects on relative perceived drinking per analysis group (means) at baseline (T1), 1-month posttest (T2), and 6-month follow-up (T3).

Additionally, absolute perceived drinking risk was higher for those who used a cue reminder in addition to the original Drinktest (B=0.27, *P*<.001) than for those who did not (see Figure 4). However, both the cue reminder (B=–0.13, *P*=.04) and prototype feedback (B=–0.11, *P*=.04) resulted in a lower relative drinking perception than when these strategies were not received in addition to Drinktest (see Figure 5). Medium-to-large effect sizes were found. The nonsignificant prototype alteration × cue reminder interaction for the secondary outcomes showed that combining the strategies did not produce an extra effect beyond the separate strategies.

### Analyses With Complete Cases Only

Finally, the analyses were repeated including full cases only (ie, without LOCF). Similar patterns of results were found as when the LOCF method was applied, albeit the effect of the cue reminder on relative perceived drinking became nonsignificant (*P*=.08). The effect of prototype alteration (in addition to Drinktest) on drinking behavior became nonsignificant.

## Discussion

### Overview

An online randomized controlled trial showed that prototype alteration and a cue reminder usage can be useful strategies to complement an existing tailored intervention (Drinktest) in reducing alcohol consumption. Although all conditions showed reductions in alcohol consumption and willingness, and increased intention to reduce drinking over a period of 6 months, reductions in alcohol consumption were higher among people who had received the prototype alteration or a cue reminder in addition to the original Drinktest compared to those who did not. The combination of the cue reminder and prototype alteration did not enhance the effect of either of the independent strategies. Importantly, participants in all conditions equally appreciated the intervention, but dropout was lower for participants who received the prototype alteration and/or cue reminder in addition to Drinktest than for participants who received the original Drinktest only.

Regarding the effect of the prototype alteration strategy, the reduced drinking levels that were found were expected. It is plausible that distancing from heavier drinking prototypes (eg, drunk and heavy drinker prototypes) [52,53] was at play, so that corresponding negative characteristics of excessive drinking were avoided (see also [33]), which may have led individuals to perceive their personal risk as lower than for others (which corresponds with this significant effect). This explanation seems to be supported by the finding that participants’ positive self-characterization increased over time (based on prototypical characteristics). It may also be that individuals changed their unhealthy behavior to feel good and positive about themselves (eg, [60]) and may be motivated to engage in self-consistent behavior and, thus, may feel less at risk than others.

The results show that cue reminders may be an effective strategy in addition to an existing intervention such as Drinktest, and that the type of cue that we provided is feasible (ie, silicone bracelet). Our study adds to the knowledge of testing the effect of cue reminders on drinking behavior [43-45] by applying it in a real-life setting (ie, participants used the cue in their own environment and aimed at self-regulation). The cue was directly linked to reducing drinking behavior and may have inhibited the urge to drink. However, although participants generally wore or used their cue frequently, they were only reasonably aware of it. Conditions did not differ in perceived attempts to reduce their drinking, but participants that received both the cue and prototype strategies (combination) contemplated more on the intervention than those who received the original Drinktest only. This may imply that rather than functioning through their salience as previously proposed, the cue reminder may have functioned through its presence in the context instead [44]. Finally, usage of the cue in addition to Drinktest was associated with changes in drinking behavior and absolute drinking risks rather than intentions. It could be that, as would be expected, the cue has reminded the participant to seize opportunities to act rather than that it changed intentions or willingness.

The interaction of prototype alteration and cue reminders did not produce an extra effect beyond the separate effects of the 2 strategies. It suggests that both strategies have an independent effect on drinking behavior, but that there is no synergistic effect by combining them. Thus, for those effects that were significant for both strategies, both may be effective but by separate means. Perhaps the link between the characteristics to be achieved and the cue reminder should have been stronger. It could be that the characteristics were already salient in the prototype alteration and hence no additional benefit of cue reminders may have occurred. Or it may be that a cue reminder does not support remembering an abstract construct such as “achievable personal characteristics” but does support the remembrance of concrete implementation intentions and action plans. To our knowledge, a bracelet as a cue reminder has not been used as a means to help decrease drinking behavior. It is conceivable that another type of cue (eg, text messages) may have a different but additional effect on the prototype alteration. Future research could shed light on this possibility.

### Limitations

The following study limitations must be addressed before discussing the implications. First, dropout was large and the sample largely consisted of Western participants. However, it is unlikely that selection based on ethnicity would have changed the results because non-Western and Western samples have been found to show similar drinking behavior in the Netherlands [2] and the analyses were corrected for ethnicity. In addition, comparison of analyses in which LOCF was applied and analyses including the full cases sample produced the same pattern of results, which may indicate that a selection bias is likely to have been limited. Moreover, it is unclear whether demand effects may have played a role, which may have caused the skewed distribution across the conditions. Also, results often only remained significant in the short term. Altogether, the results should be interpreted with caution and generalizability may be decreased due to the larger dropout among specific groups. Furthermore, the results were based on self-report. However, we do not think that underreporting was a problem because of the removal of outliers based on the MAD method in the measurements. In addition, the prototype alteration and cue effects that were found in addition to Drinktest can be partly explained by the addition of action plans, although they both had unique contributions to the outcomes. The effects are meaningful and are generally consistent with expectations. Finally, tailored feedback was provided at baseline only. Although the results span a period of 6 months, future studies could determine whether feedback at several measurement points would improve these findings.

### Implications and Future Research

The findings suggest the following implications and future directions. First, our findings support earlier suggestions that future interventions may benefit from providing relevant prototypes to be achieved and avoided [29] and to tailor prototypical characteristics according to the individuals’ relevance [53]. Heavier drinking prototypes (eg, heavy drinker, drunk) [52] could be relevant prototypes to be distanced from by accentuating the attributed negative characteristics [33], and the moderate drinker prototype could be encouraged to assimilate with [29] by accentuating the achievability of its positive characteristics if alcohol consumption were reduced. Thus, in the case of experienced drinkers, modifying the valence of prototypes could prove worthwhile and the effect of prototypes on drinking behavior could be overcome by implementation intentions or action plans (see also [41]).

Second, the bracelet had the advantage of being self-regulated by participants and that it can be effective even when alcohol is already consumed [43,44]. However, only limited knowledge is available on the effectiveness of different types of cue reminders. Future research should determine which type of cue reminder is most effective and how to make individuals more aware of the cue. Future research also needs to be aware of the different mechanisms influencing the effect of cue reminders.

Third, it may be important for future interventions to complement the strategies with messages that make people aware of their drinking behavior and that especially informs excessive drinkers about the consequences of their behavior as was done by the original Drinktest [15]. However, future research is necessary to further our understanding of how to optimize prototype alteration and cue reminders as strategies.

## Multimedia Appendix 1

Examples of tailored feedback.

## Multimedia Appendix 2

CONSORT-EHEALTH checklist V1.6.2 [61].

## Abbreviations

ES(f): effect size f
LOCF: last observation carried forward
MAD: median absolute deviation
PWM: Prototype Willingness Model
QFV: Quantity-Frequency-Variability
TPB: Theory of Planned Behavior
