# Supplementary material for: Drinker Prototype Alteration and Cue Reminders as Strategies in a Tailored Web-Based Intervention Reducing Adults’ Alcohol Consumption: Randomized Controlled Trial
Source: J Med Internet Res. 2015 Feb 4;17(2):e35. doi: 10.2196/jmir.3551 (PMC4342749; doi:10.2196/jmir.3551)
Supplement: Supplementary file 1 [file jmir_v17i2e35_app1.pdf]

Multimedia appendix 1. Examples of tailored feedback

Condition	Example
Original Drinktest	Perceived susceptibility: "With regard to your health, you do drink too much, although you reported not to think so…"<br><br>Intentions: "You intend to reduce your alcohol consumption within half a year. That's a good idea, because your current behaviour can cause you permanent health damage. To avoid damage, you will have to reduce your drinking. But why not in the short-term?"<br><br>Attitude – benefits: "You said alcohol makes you feel more relaxed. … But note that only small amounts of alcohol can work relaxing. After that, alcohol will make you feel more tense and anxious. By reducing your alcohol consumption you will probably feel more relaxed, actually."<br><br>Attitude – downsides: "… Your alcohol use is not good, but actually unhealthy (bad) for your liver. If you'd reduce your alcohol use, your liver can recover. This can reduce potential health complaints and avoids permanent damage."<br><br>Temptations: "It is tempting for you to drink alcohol when you are having a fight. You may know that alcohol can worsen fights. … It will be better to be sober to solve the fight. Take the time and find out what it really is about …"
Prototype alteration	Example of reflection on some negative and positive characteristics:<br>"Your answers show you'd like to be [social, irresponsible, uncontrolled, and spontaneous]. The characteristics of [social and spontaneous] correspond best with drinking moderately. Your peers generally value these characteristics the most and find those important. The norm of drinking moderately is …"<br><br>Example of reflection on drinking behaviour:<br>"You have consumed a total of [36] glasses of alcohol. As you can see, this exceeds the norm of drinking moderately. …<br>Your peers generally will regard excessive drinking as annoying, uncontrolled, volatile, and insecure. They generally value these characteristics as negative and undesirable."<br><br>Example of reflection on intention:<br>"You intend to reduce your alcohol use within a month. That's great, because, if you drink less, you will be more likely to appear more positively and be positively valued!"
Cue reminder	Example of reflection on goal setting and encouragement to use a cue reminder:<br>"… You reported to doubt whether you'd like to reduce your alcohol use. … Yet, you have set the goal to quit drinking. A cue reminder can help you remember your goal and the information provides by Drinktest …"<br><br>Example of instruction to remember one's goal:<br>"Every time that you are aware of the bracelet think of your goal to quit."
Combined	"Every time that you are aware of your [bracelet] think of your goal to [quit

	drinking] and the positive characteristics you can achieve.”
