# Supplementary material for: Drinker Prototype Alteration and Cue Reminders as Strategies in a Tailored Web-Based Intervention Reducing Adults’ Alcohol Consumption: Randomized Controlled Trial
Source: J Med Internet Res. 2015 Feb 4;17(2):e35. doi: 10.2196/jmir.3551 (PMC4342749; doi:10.2196/jmir.3551)
Supplement: Supplementary file 2 [file jmir_v17i2e35_app2.pdf]

# CONSORT-EHEALTH Checklist V1.6.2 Report

**(based on CONSORT-EHEALTH V1.6), available at [http://tinyurl.com/consort-ehealth-v1-6].**

3551

**Date completed**

5/18/2014 9:02:21

**by**

van Lettow

Drinker prototype alteration and cue reminders as strategies in a tailored web-based intervention reducing adults' alcohol consumption: an RCT

## TITLE

**1a-i) Identify the mode of delivery in the title**

"strategies in a tailored web-based intervention"

**1a-ii) Non-web-based components or important co-interventions in title**

- In title: "cue reminders as strategies"
- The cue reminder is one of the components of the intervention. The tailored feedback is web-based, the actual cue reminder is used in real life (i.e., a silicon bracelet).

**1a-iii) Primary condition or target group in the title**

In title: "adults"

## ABSTRACT

**1b-i) Key features/functionalities/components of the intervention and comparator in the METHODS section of the ABSTRACT**

- "A web-based randomized controlled trial tested two strategies (i.e., prototype alteration and cue reminders) within an existing online personalized feedback intervention ('Drinktest') aiming at reducing adults' excessive drinking"
- "Excessive drinking adults (N=2634) were randomly assigned to four conditions"
- "one and six months follow-up"
- "Differences in drinking behavior, intentions and behavioral willingness (i.e., primary outcomes) were assessed by means of longitudinal multilevel analyses using a 'last observation carried forward' method."

**1b-ii) Level of human involvement in the METHODS section of the ABSTRACT**

- "Excessive drinking adults (N = 2634) were randomly assigned to four conditions: original Drinktest, Drinktest plus prototype alteration, Drinktest plus cue reminder, and Drinktest plus prototype alteration and cue reminder. "

**1b-iii) Open vs. closed, web-based (self-assessment) vs. face-to-face assessments in the METHODS section of the ABSTRACT**

- "Participants were recruited online and through printed materials."
- "Measures were based on self-reports."

**1b-iv) RESULTS section in abstract must contain use data**

- "N=2634)
- "All conditions showed reductions in drinking behavior and willingness to drink, and in increased intentions to reduce drinking" (regarding the primary outcomes)

**1b-v) CONCLUSIONS/DISCUSSION in abstract for negative trials**

N/A, the study found changes in behavior, as expected.

## INTRODUCTION

**2a-i) Problem and the type of system/solution**

- "It is important to further our understanding of how to reduce excessive drinking"
- "A meta-analysis showed that, among the interventions that were based on the goal-striving Theory of Planned Behavior (TPB) components, only half were found to guide changes in intentions and two-third guided changes behavior, and only small effect sizes were produced."
- "The present study addresses these issues by examining the effect of two intervention strategies that could potentially help enhancing the effect of an existing online (i.e., web-based) tailored intervention, Drinktest.nl: prototype alteration and cue reminders."

**2a-ii) Scientific background, rationale: What is known about the (type of) system**

- "In the Netherlands, 12.9% of the general population engages in weekly binge drinking"
- "A majority of interventions have targeted drinking behavior assuming that behavior is intentional."
- "behaviors may occur without intentions or even when having intentions not to do so"
- "online self-help interventions have been found to be effective in reducing adults' drinking behavior in the general population with an effect up to 6 or 9 months ... However, single-session interventions, such as Drinktest.nl, have been found to produce small effect sizes only"
- "In sum, the studies suggest that a significant proportion of intentions and behavior remains unexplained and that the effectiveness of interventions can be improved."
- "Experiments and intervention studies revealed that prototype alteration was effective in (1) postponing the onset of drinking among children aged 10–12 years old with an effect up to two years, ..."
- "Although prototype alteration has been applied to alcohol use, only few interventions aimed at reducing excessive drinking based on dual-process models (PWM) have been applied to young adults (usually incorporating only university students), and results have been mixed ... there are no such interventions for the general adult population"
- "Cue reminders can support enactment of intentions as they can unconsciously prompt self-enhancing or self-protecting opportunities"

## METHODS

**3a) CONSORT: Description of trial design (such as parallel, factorial) including allocation ratio**

- "This study examined whether prototype alteration and provision of a cue reminder can be useful strategies to enhance the effectiveness of an existing online (i.e., web-based) tailored intervention, i.e., Drinktest.nl."
- "Expected was that (a) prototype alteration may intrinsically motivate people to drink less, (b) cue reminders may strengthen the salience of alcohol reduction goals, and (c) the combination of prototype alteration and a cue reminder may increase the salience and intrinsic motivation of alcohol reduction goals."

**3b) CONSORT: Important changes to methods after trial commencement (such as eligibility criteria), with reasons**

There were no changes after trial commencement.

**3b-i) Bug fixes, Downtimes, Content Changes**

The intervention has only been offline a couple of hours and should not have influenced the results.

**4a) CONSORT: Eligibility criteria for participants**

- "Eligible participants were individuals aged 18 or over engaging in excessive drinking: exceeding 14 and 21 or more glasses of alcohol per week or drinking 4 and 6 glasses or more per occasion for women and for men, respectively. This norm was set by the original Drinktest and was thus left unchanged."

**4a-i) Computer / Internet literacy**
The majority of Dutch adults (18+) have access to the internet. Computer/internet literacy should not be an important issue for the purpose of this study.
**4a-ii) Open vs. closed, web-based vs. face-to-face assessments:**
"Participants were recruited online and by printed materials from September 2012 till June 2013. The website of www.drinktest.nl was also easily accessible by online search engines."
**4a-iii) Information giving during recruitment**
"At start (T1), participants read the study information and were told that the existing Drinktest was being evaluated. Importantly, it was explicitly stated that participants did not have to commit themselves to reducing their alcohol consumption. Participants were then asked to sign the online informed consent form."
**4b) CONSORT: Settings and locations where the data were collected**
- "A randomized controlled trial was conducted in the Netherlands"
- "September 2012 till June 2013"

**4b-i) Report if outcomes were (self-)assessed through online questionnaires**
"All questions were self-administered and data was collected online."
**4b-ii) Report how institutional affiliations are displayed**
- The Trimbos-Institute had placed a banner on their website to recruit participants.
- Participants were told that the Erasmus MC collaborated with Mentalshare in evaluating the Drinktest (including each logo).

**5) CONSORT: Describe the interventions for each group with sufficient details to allow replication, including how and when they were actually administered**
**5-i) Mention names, credential, affiliations of the developers, sponsors, and owners**
- "Drinktest was developed by the Netherlands Institute for Health Promotion and Disease Prevention (NIGZ)."
- Drinktest is now owned by Mentalshare
- In conflicts of interest: "The second author is scientific director of Vision2Health, a company that licenses evidence based, innovative, computer-tailored health communication tools."
- "This research was supported by a grant of project no. 121020020 from ZonMW, Netherlands Organization for Health Research and Development"

**5-ii) Describe the history/development process**
- "Measures from the original Drinktest were left unchanged and were extended by items regarding demography, willingness, prototypes, cue reminder, and process evaluation."
- The intervention was piloted and tested before commencement.

**5-iii) Revisions and updating**
The intervention was first piloted. Comments were used to improve the intervention content. After this, the intervention was launched and left unchanged. Tailored feedback was provided at baseline and as a result, the intervention has not been changed after its evaluation.
**5-iv) Quality assurance methods**
The strategies have been tested thoroughly before commencement.
**5-v) Ensure replicability by publishing the source code, and/or providing screenshots/screen-capture video, and/or providing flowcharts of the algorithms used**
- The supplementary multimedia appendix 1 provides examples of the feedback. There are too many examples and combinations of feedback possible to provide all texts.
- A flow chart of the intervention is provided so that it is clear which questions/feedback is provided per condition.

**5-vi) Digital preservation**
The original Drinktest is owned by Mentalshare and is still in use. The intervention version is offline but accessible for our research team.
**5-vii) Access**
- Participants could find the intervention through our printed and online advertisements and access the intervention online only.
- "A total of 50 vouchers worth €50 were distributed (by means of a raffle) as incentive."
- "Participants were invited by email to participate in the two follow-up measurements and received reminders if necessary"

**5-viii) Mode of delivery, features/functionalities/components of the intervention and comparator, and the theoretical framework**
- "Feedback was derived from a computer program linking each possible combination of responses with an appropriate message. Feedback was not provided during the second and third measurement."
- In-dept information on the content is provided per condition, e.g., "participants in the prototype condition received feedback regarding prototype alteration (see measures and Figure 1, and see Appendix A for examples) tailored to gender, drinking behavior (also including normative feedback), intentions and prototypical self-characterization."
- The Theory of Planned Behavior and the Prototype/Willingness Model were the underlying theoretical frameworks.
- "... These messages reflected on personal drinking levels in comparison to the Dutch norm and peers' drinking behavior, the correctness of their absolute and relative perceived susceptibility for health risks due to their alcohol consumption, intentions, temptations (e.g., coping with fights), correctness of positive effects of alcohol (e.g., whether alcohol helps to sleep better), and correctness of negative effects of alcohol (e.g., consequences for the liver and heart)."

**5-ix) Describe use parameters**
Feedback was only provided once. We do not have information on the time spent to finish modules, except for the time spent in the pilot.
**5-x) Clarify the level of human involvement**
Participants did not receive any assistance besides the content of the intervention.
All authors contributed to the content of the added modules (added to the original Drinktest). Two persons from an ICT company (E-Vision) made it possible for us to add the feedback and modules.
**5-xi) Report any prompts/reminders used**
- The cue reminder was meant to remind people of the intervention and was part of the strategies of behavioral change.
- Participants were invited by email to participate in the two follow-up measurements and received reminders if necessary.

**5-xii) Describe any co-interventions (incl. training/support)**
N/A, participants only were offered the intervention and were randomized among one of the four conditions.
**6a) CONSORT: Completely defined pre-specified primary and secondary outcome measures, including how and when they were assessed**
- the flow charts provides information which questions were addressed when.
- the tables, method section, and results section provide information and outcomes for the primary and secondary outcomes. The method section specifies exactly which items were assessed.

For instance: "To assess intentions, the item was framed by Drinktest in behavioral stages ranging from (1) 'I do not plan to reduce my alcohol consumption' to (5) I have reduced my alcohol consumption more than half a year ago'. This single item was treated as a continuous variable." This item is placed under the heading "Primary outcome measures".

**6a-i) Online questionnaires: describe if they were validated for online use and apply CHERRIES items to describe how the questionnaires were designed/deployed**
All items that were used in the original Drinktest have been used and left unchanged. Items (and feedback) added for our study purposes were piloted online and were based on our previous online research.
**6a-ii) Describe whether and how "use" (including intensity of use/dosage) was defined/measured/monitored**
N/A. If participants entered the intervention, they were regarded as "using" the program.
**6a-iii) Describe whether, how, and when qualitative feedback from participants was obtained**
"Participants reported on their appreciation of the intervention at T2 answering to the statement 'the information and advice of Drinktest.nl were…', ranging from (1) 'I disagree' to (7) 'I agree' regarding reliability, novelty, being informative, ease of understanding, personal relevance, persuasiveness, enjoyability, and usefulness (α=.86).
At T2 and T3, all participants were asked, regarding the past four weeks, (a) how aware they had been of their alcohol use, (b) how often they had contemplated on the intervention's feedback, and (c) their perception of having tried to reduce their alcohol consumption."
**6b) CONSORT: Any changes to trial outcomes after the trial commenced, with reasons**
N/A. Outcomes were specified beforehand and have been specified in the manuscript.
**7a) CONSORT: How sample size was determined**
**7a-i) Describe whether and how expected attrition was taken into account when calculating the sample size**
- "longitudinal multilevel analyses ('mixed models', Table 3) were performed using the 'last observation carried forward' (LOCF) method (a) to account for drop-out"
- "Finally, for sensitivity purposes the analyses were repeated for complete cases only."
- "Importantly, we used the median absolute deviation (MAD) to detect outliers. MAD was applied because it is more robust to outliers than the standard deviation"
**7b) CONSORT: When applicable, explanation of any interim analyses and stopping guidelines**
N/A
**8a) CONSORT: Method used to generate the random allocation sequence**
- "participants were randomly assigned (computerized) to one of four conditions"
- "Feedback was derived from a computer program linking each possible combination of responses with an appropriate message."
**8b) CONSORT: Type of randomisation; details of any restriction (such as blocking and block size)**
Simple randomization was conducted by computer
**9) CONSORT: Mechanism used to implement the random allocation sequence (such as sequentially numbered containers), describing any steps taken to conceal the sequence until interventions were assigned**
Simple randomization was conducted by computer, concealed for participants.
**10) CONSORT: Who generated the random allocation sequence, who enrolled participants, and who assigned participants to interventions**
Participants were randomized by computer, allocating them to one of the four conditions.
**11a) CONSORT: Blinding - If done, who was blinded after assignment to interventions (for example, participants, care providers, those assessing outcomes) and how**
**11a-i) Specify who was blinded, and who wasn't**
Participants were unaware of their allocation to one of the four conditions.
**11a-ii) Discuss e.g., whether participants knew which intervention was the "intervention of interest" and which one was the "comparator"**
Participants were unaware of their allocation to one of the four conditions and thus did not know that there was a comparator.
**11b) CONSORT: If relevant, description of the similarity of interventions**
All conditions received the original Drinktest modules. Three conditions received one or two additional strategies.
**12a) CONSORT: Statistical methods used to compare groups for primary and secondary outcomes**
- "longitudinal multilevel analyses ('mixed models', Table 3) were performed using the 'last observation carried forward' (LOCF) method (a) to account for drop-out and (b) because of the nested design (measurements, i.e., time, were nested in individuals)."
**12a-i) Imputation techniques to deal with attrition / missing values**
- "performed using the 'last observation carried forward' (LOCF) method (a) to account for drop-out"
**12b) CONSORT: Methods for additional analyses, such as subgroup analyses and adjusted analyses**
- "The analyses were also corrected for potential significant differences between conditions at baseline (see Table 1)."
- "for sensitivity purposes the analyses were repeated for complete cases only."
- "Intervention analyses were corrected for age and educational level because these were significantly different between conditions at baseline."

## RESULTS

**13a) CONSORT: For each group, the numbers of participants who were randomly assigned, received intended treatment, and were analysed for the primary outcome**
- "The resulting final sample consisted of 2634 eligible participants"

Flow chart:
- "860 in original Drinktest"
- "660 in prototype condition"
- "597 in cue condition"
- "517 in combined condition"

**13b) CONSORT: For each group, losses and exclusions after randomisation, together with reasons**
- Reasons for losses to follow-up are unfortunately omitted to us.
- "1374 lost to follow-up"

**13b-i) Attrition diagram**
"Figure 2. Flow chart of participants"
This figures presents the attrition diagram.
**14a) CONSORT: Dates defining the periods of recruitment and follow-up**
- "Participants were recruited online and by printed materials (posters and newspaper advertisements) from September 2012 till June 2013."
- "A one- and six-months follow-up measurement was conducted (post-intervention: T2, T3)."

**14a-i) Indicate if critical "secular events" fell into the study period**
N/A
**14b) CONSORT: Why the trial ended or was stopped (early)**
The intervention was online for a longer period than planned to be able to collect more data. It was not stopped earlier.
**15) CONSORT: A table showing baseline demographic and clinical characteristics for each group**

- "Table 1 presents the baseline characteristics of participants overall and per condition."
**15-i) Report demographics associated with digital divide issues**
"Table 1 presents the baseline characteristics of participants overall and per condition."

No information on internet/computer literacy is available to us.
**16a) CONSORT: For each group, number of participants (denominator) included in each analysis and whether the analysis was by original assigned groups**
**16-i) Report multiple "denominators" and provide definitions**
Analyses were only by original assigned groups. Each table with outcomes provides the N per condition and the overall N.
**16-ii) Primary analysis should be intent-to-treat**
- "longitudinal multilevel analyses ('mixed models', Table 3) were performed using the 'last observation carried forward' (LOCF) method"
- "for sensitivity purposes the analyses were repeated for complete cases only."

Thus, LOCF was used instead.
**17a) CONSORT: For each primary and secondary outcome, results for each group, and the estimated effect size and its precision (such as 95% confidence interval)**
Table 3 presents all outcomes, including 95% confidence intervals per condition and overall.
**17a-i) Presentation of process outcomes such as metrics of use and intensity of use**
- "Second, the appreciation of the intervention was assessed. The original (M=4.85, SD=.96) and extended Drinktest (combining the three extended conditions; M=4.88, SD=1.12) did not differ in their intervention evaluations (F(1, 802)=0.06, P=.81). Both Drinktest versions were rated equally interesting, new, informative, understandable, personally relevant, persuasive, enjoyable, and useful. The results were similar across all four conditions."
- "Furthermore, participants in the cue and combination conditions were found to remember their chosen cue reminder correctly (94.1%). The majority (61.4%) reported to use or wear their cue reminder frequently. The awareness of the cue was reasonable (M=3.27, SD=2.11)."
**17b) CONSORT: For binary outcomes, presentation of both absolute and relative effect sizes is recommended**
N/A, all outcomes were continuous.
**18) CONSORT: Results of any other analyses performed, including subgroup analyses and adjusted analyses, distinguishing pre-specified from exploratory**
- No subgroup analyses were performed.
- "analyses are corrected for differences at baseline (i.e., age and educational level) and include all three measurements"
**18-i) Subgroup analysis of comparing only users**
N/A
**19) CONSORT: All important harms or unintended effects in each group**
There are no potential harms in participating in any of the conditions. Increased drinking due to the intervention is very unlikely.
**19-i) Include privacy breaches, technical problems**
N/A. Data was treated with great care and anonymously.
**19-ii) Include qualitative feedback from participants or observations from staff/researchers**
An open item at the end of each measurement (i.e., wave) enabled participants to note any other thoughts besides the evaluation questions.

## DISCUSSION

**20) CONSORT: Trial limitations, addressing sources of potential bias, imprecision, multiplicity of analyses**
**20-i) Typical limitations in ehealth trials**
Participants were blinded regarding their allocation to conditions. They were told that Drinktest was being evaluated and are unlikely to know which primary and secondary outcomes were tested, also since there were many questions.
**21) CONSORT: Generalisability (external validity, applicability) of the trial findings**
**21-i) Generalizability to other populations**
The results are generalizable to the Dutch general population. It is unclear whether other Western countries would result in the same findings.
**21-ii) Discuss if there were elements in the RCT that would be different in a routine application setting**
It is unclear whether several measurements that provide feedback will improve the present findings. Previous research is mixed regarding the enhancing effect, because multiple sessions can be related to reduced uptake.
**22) CONSORT: Interpretation consistent with results, balancing benefits and harms, and considering other relevant evidence**
**22-i) Restate study questions and summarize the answers suggested by the data, starting with primary outcomes and process outcomes (use)**
"An online randomized controlled trial showed that prototype alteration and a cue reminder usage can be useful strategies to complement an existing tailored intervention ('Drinktest') in reducing alcohol consumption. Specifically, although all conditions showed reductions in alcohol consumptions and willingness and increased intentions to reduce drinking over a period of 6 months, reductions in alcohol consumption were higher among people who had received the prototype alteration or a cue reminder in addition to the original Drinktest compared to those who did not. The combination of the cue reminder and prototype alteration did not enhance the effect of either of the independent strategies. Importantly, participants in all conditions equally appreciated the intervention, but drop-out was lower for participants who received the prototype alteration and/or cue reminder in addition to Drinktest than for participants who received the original Drinktest only."
**22-ii) Highlight unanswered new questions, suggest future research**
- "Although the results regard a period of six months, future studies could determine whether feedback at several measurements will improve the present findings."
- "our findings support earlier suggestions that future interventions may benefit from providing relevant prototypes to be achieved and avoided and to tailor prototypical characteristics according to the individuals' relevance."
- "Future research should determine which type of cue reminder is most effective and how to make individuals more aware of the cue. Future research also needs to be aware of the different mechanisms influencing the effect of cue reminders."

## Other information

**23) CONSORT: Registration number and name of trial registry**
"Trial registration ID: NTR 4169 (http://www.trialregister.nl)."
**24) CONSORT: Where the full trial protocol can be accessed, if available**
Mentalshare is owner of the original Drinktest and its protocol.
**25) CONSORT: Sources of funding and other support (such as supply of drugs), role of funders**
"This research was supported by a grant of project no. 121020020 from ZonMW, Netherlands Organization for Health Research and Development."
**X26-i) Comment on ethics committee approval**
"Ethical approval by an independent ethics committee (ref. no. MEC-2010-112)"

**x26-ii) Outline informed consent procedures**
- "At start (T1), participants read the study information and were told that the existing Drinktest was being evaluated. Importantly, it was explicitly stated that participants did not have to commit themselves to reducing their alcohol consumption. Participants were then asked to sign the online informed consent form. In case participants declined to participate, they could leave the browser or receive the old Drinktest."
- Participants received standard information regarding their anonymity and their possibility to quit the program whenever they wished.

**X26-iii) Safety and security procedures**

The original Drinktest provides phone numbers and websites. Also, the emailadres of the lead author was provides to answer any questions.

**X27-i) State the relation of the study team towards the system being evaluated**

"The second author is scientific director of Vision2Health, a company that licenses evidence based, innovative, computer-tailored health communication tools. All other authors declare that they have no conflicts of interest."
